# Study of the Applicability Domain of the QSAR Classification Models by Means of the Rivality and Modelability Indexes

**DOI:** 10.3390/molecules23112756

**Published:** 2018-10-24

**Authors:** Irene Luque Ruiz, Miguel Ángel Gómez-Nieto

**Affiliations:** Department of Computing and Numerical Analysis, Campus Universitario de Rabanales, Albert Einstein Building, University of Córdoba, E-14071 Córdoba, Spain; mangel@uco.es

**Keywords:** QSAR, classification model, applicability domain, rivality index, modelability index

## Abstract

The reliability of a QSAR classification model depends on its capacity to achieve confident predictions of new compounds not considered in the building of the model. The results of this external validation process show the applicability domain (AD) of the QSAR model and, therefore, the robustness of the model to predict the property/activity of new molecules. In this paper we propose the use of the rivality and modelability indexes for the study of the characteristics of the datasets to be correctly modeled by a QSAR algorithm and to predict the reliability of the built model to prognosticate the property/activity of new molecules. The calculation of these indexes has a very low computational cost, not requiring the building of a model, thus being good tools for the analysis of the datasets in the first stages of the building of QSAR classification models. In our study, we have selected two benchmark datasets with similar number of molecules but with very different modelability and we have corroborated the capacity of the predictability of the rivality and modelability indexes regarding the classification models built using Support Vector Machine and Random Forest algorithms with 5-fold cross-validation and leave-one-out techniques. The results have shown the excellent ability of both indexes to predict outliers and the applicability domain of the QSAR classification models. In all cases, these values accurately predicted the statistic parameters of the QSAR models generated by the algorithms.

## 1. Introduction

Research on the applicability domain (AD) of Quantitative Structure-Activity Relationship (QSAR) models has caught the attention of the Chemometric community in the last years [1,2,3,4,5,6,7,8]. From the publication of the OECD report [9] describing the principles for the validation of QSAR models, several proposals have been published with the aim of determining the AD of QSAR models.

According to the OECD guiding principles, a QSAR model should have: (i) a defined endpoint, (ii) an unambiguous algorithm, (iii) a defined domain of applicability, (iv) appropriate measures of goodness-of-fit, robustness, and predictivity, and (v) a mechanistic interpretation where possible [9,10].

The AD concept can be applied in different ways, depending on the type of QSAR model and modeling approach [11]. Some approaches for defining the AD are based on similarity analysis [12] considering the AD as a measurement aimed to estimate the uncertainty in the prediction of a particular molecule based on how similar it is to the molecules used in the building of a model. Thus, AD can be defined as a distance measurement between all the molecules used in the building of the model and, also, the distance between the molecules of the training and external sets.

In summary, the goal of the AD approaches is to estimate the prediction reliability for each modeled compound individually. Using this information, it would be possible to estimate the accuracy of prediction for an arbitrary data set regardless of its similarity to the set used to validate the model [13].

QSAR studies can assess the accuracy of predictions in different ways [14,15,16]. The simple ones try to distinguish reliable versus non reliable predictions, assuming that the accuracy of prediction of molecules, which are inside a space of descriptors covered by the training set, is similar to the estimated accuracy of the model.

Due to the data representation of the molecules being, generally, based on molecular descriptors as variables, the applicability domain has been defined by different methods: the value ranges of these variables, the value ranges of the principal components of these variables, the optimal prediction space (TPKAT), geometric methods, probabilistic density distribution methods, distance based methods, etc. [17,18]

All of these methods are based on the QSAR principle of similarity, so a molecule falls within of the AD of a model if in the training set there is, at a closer distance, similar molecules with more similar activity than any other dissimilar molecule of similar activity.

Most of the proposals used to measure the AD are based on the distance based methods (DM) considering that a molecule is inside the AD when the distance from this molecule to the distance of the training set is lower than a predefined threshold. 

Carrió et al. [19] have proposed a method (ADAN) based on the calculation of six measurements: (i) the distance between the query compound and the centroid of the training set, (ii) the distance between the query compound and the closest compound in the training set, (iii) the distance to model (DModX), (iv) the difference between the predicted biological property and the average biological property in the training set, (v) the difference between the predicted biological property and observed biological value for the closest compound in the training set, (vi) SDEP (standard deviation error of the predictions) of 5% of the closest compounds in the training sets to the query compound.

Although the method contains no tunable parameters and can work unsupervised, producing reasonable estimations of the prediction errors in highly unfavorable conditions, it requires a high number of calculations and the execution of algorithms for the building of the models and calculations of the parameters used for the predictions. 

Yun et al. [20] have proposed to define the AD by means of population analysis (PA) strategy, including model population analysis (MPA) and approach population analysis (APA). MPA employed bounding box, Mahalannobis distance and K-nearest neighbors methods to define AD with a large amount of sub-datasets derived from training set. APA was used to get a union of all results generated by the AD approaches to give a consensus list of samples as falling outside the AD.

Liu and Wallqvist [2] have proposed the use of the fusion of random forest (RF) and variable nearest neighbor method (v-NN) for the building of classification models of Ames mutagenecity benchmark dataset. In this proposal, authors show the advantages of extended connectivity fingerprint (ECFP) for the dataset representation. The predictions of the class belonging to each molecule of the dataset is carried out by means of the following expression:

(1) $$\;y=\frac{\sum_{i=1}^{v}y_{i}e^{-{\left(\frac{d_{i}}{h}\right)}^{2}}}{\sum_{i=1}^{v}y_{i}e^{-{\left(\frac{d_{i}}{h}\right)}^{2}}},\;d\leq d_{0}\;$$

where *d_i_* denotes the Tanimoto distance between a target molecule for which a prediction is made and molecule *i* of the training set, *y_i_* denotes the experimentally measured value of molecule *i*, *v* denotes the total number of training set molecules satisfying the condition *d_i_ ≤ d_0_*, *h* is a smoothing factor which dampens the distance penalty, and *d_0_* is a Tanimoto-distance threshold beyond which two molecules are not considered sufficiently similar to be included in the average.

Therefore, this approach has the lack of needing to adjust two parameters: the molecular structural similarity threshold, determining the threshold of the AD and the smoothing factor, although the authors reach a clear improvement in the accuracy with a high coverage.

Roy et al. [21,22] proposed a standardization approach to define the outliers in the training set and identifying the compounds that reside outside the AD in the test set. Sushko et al. [18] demonstrated that the DMs based on an ensemble (consensus) model provide systematically better performance than other DMs. In this paper, the authors use 30 QSAR models being capable of identifying 30–60% of molecules having an accuracy of prediction similar to the inter-laboratory accuracy of the Ames test, which is estimated to be 90%, proposing that the in-silico predictions can be used to halve the cost of experimental measurements by providing a similar prediction accuracy. Authors used different MD calculations for the molecules of the dataset to calculate the accuracy in the predictability of the molecule activity: the standard deviation of the predictions (STD, leverage, correlation, concordance, CLASS-LAG, etc.).

CLASS-LAG method provides a simple and clear measure of the AD. This method proposed by Manallack et al. [23] is used for binary datasets (molecules belonging to two classes: −1, +1), and with those machine learning algorithms providing continuous (not discrete) results of the predictions. For each molecule of the dataset a continuous value in the interval [−1, +1] is obtained as follows:

(2) $$\;d_{CLASS-LAG}\left(J\right)=\;\min\left\{\left|-1-y\left(J\right)\right|,\left|1-y\left(J\right)\right|\right\}\;$$

where: *J* represents the molecules of the dataset and *y*(*J*) is the prediction value of *J* obtained by the machine learning algorithm used in the building of the model.

Thus, the distance between *d_CLASS-LAG_*(*J*) value for the molecule *J* and the class (−1 or +1) to which *J* belongs is a measurement of the accuracy of the predictability of *J*.

Despite the large number of DM methods proposed for the calculation of AD and the goodness of these methods to detect the molecules inside and outside of the AD of a model, the fault in the AD studies with DM methods is to find those molecules which are in the edge of the predictability, so different results are obtained depending of the DM method used, what different authors have proposed is to combine different DM approaches and strategies [24,25].

In this paper, we propose a simple and fast DM method for the calculation of the AD. The method does not require the building of a model, so it can be used in the first stages of the QSAR investigation. Thus, the AD calculation is independent to the machine learning used in the building of a model. The aim of our approach is to propose a simple DM measurement capable of providing the researcher, in the initial stages of the development of a QSAR model, information about the molecules of the training set, the expected results on the robustness of the model and the capability of this model to correctly predict the activity of new molecules.

Although in this paper we describe the approach for classification models, the method can be extended to regression problems. For classification problems, a commonly used measure of prediction accuracy is Correct Classification Rate (*CCR*), which is simply the percentage of compounds correctly classified by a model. *CCR* is appropriate for balanced sets, and for binary datasets sensitivity and specificity are also measures of the accuracy of the prediction. 

The method proposed can be applied to the molecules in the training and external sets (for external validation) and to calculate a global measure of the *CCR* for the training set and a local measure of the predictability of each one of the molecules of the training and external sets.

In our study, we have selected two benchmark datasets with similar number of molecules but with very different modelability. Then, we have built classification models using Support Vector Machine and Random Forest algorithms with 5-fold cross-validation and leave-one-out techniques.

The local measure of the predictability of each molecule is calculated by means of its rivality index (*RI*) [26]. The rivality index is a measure of the capacity of each molecule to be correctly classified by an algorithm. Independently of the classes existing in the dataset and the assigned label (Positive/Negative, Active/Inactive, classes 0 and 1, classes −1 and +1, etc.), the *RI* assigns values in the interval [−1, +1] to each molecule. 

Molecules with high positive values are determined as outside of the AD. Molecules with high low negative values are determined as inside of the AD. In addition, molecules with *RI* value close to zero (positive or negative) are determined as “activity borders” [26]. Thus, our proposal is capable of finding those molecules which are in the edge of the predictability and, even to determine the capacity of an algorithm to classify, and to predict, correctly/incorrectly these molecules.

Global measurements of the *RI* for the whole training and test sets allow us to calculate the modelability index of the training set and the predictability of the external set. The modelability index values calculated for the datasets have shown the different characteristics of the datasets and their different capacity of being modeled. In addition, the rivality index values for the molecules of each dataset have allowed to detect the activity cliff molecules, predicting the outliers or false positive molecules detected by the algorithms. These results allow to predict the results of the algorithm and the robustness of the built model.

Moreover, we have carried out the external validation process for these datasets in order to study the AD of the QSAR models. In this process, we have partitioned the datasets in two independent sets. The training set has been used for the building of the QSAR models with leave-one-out technique, and the external set has been used for the external validation and analysis of the AD of the built models. This process was performed three times carrying out three random partitions of each dataset.

External validation results obtained for each algorithm were compared with the rivality index values calculated for each molecule of the external set and the modelability index values obtained for the external set. This analysis has shown the excellent ability of both indexes to predict outliers and the applicability domain of the QSAR classification models. In all cases, these values accurately predict the statistic parameters of the QSAR models generated by the algorithms. The manuscript has been organized as follows: next to the Introduction. In Section 2 we describe the experimental carried out for the two datasets and we discuss the results. In the Discussion section we comment the advantages of the new DM method proposed for the analysis of the applicability domain. Finally, in Materials and Methods we have described the datasets used for the calculations and the foundations of the rivality and modelability indexes.

## 2. Results

### 2.1. Behavior of RI and CMODI Indexes, and RF and SVM Algorithms with the YES1 Dataset

Initially we have studied the behavior of RF and SVM algorithms in order to analyze the results of the models built. In this study we build the classification models using LOO and 5-folds cross-validation (CV). Using CV, we have repeated the building of the models five times. Thus, we were able to study the stability of the algorithms in the detection of outliers (false class 0 and false class 1 molecules).

**Figure 1:**
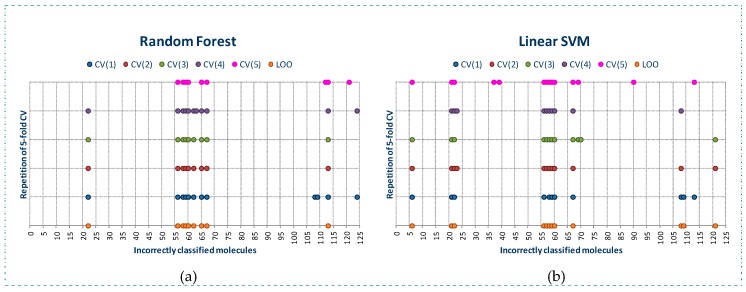
Outliers detected by RF (**a**) and SVM (**b**) in the building of classification models for YES1 dataset using LOO and 5-fold cross-validation (5 models were built).

As observed in Figure 1, RF algorithm detects a lower number of molecules incorrectly classified than SVM algorithm, although the molecules detected as incorrectly classified by RF are also detected by SVM algorithm. RF algorithm detects a lower number of molecules incorrectly classified using LOO than almost all of the models built with CV, belonging most of these molecules to class 0. We can also observe that for the five models built using CV, the molecules incorrectly classified (these molecules will be named as outliers along the manuscript) detected are practically the same, so RF is a stable classification algorithm.

The behavior of SVM is quite similar. Using LOO a lower number of outliers is detected than when using CV. However, in the five models built using CV, a different number of outliers is detected.

For both algorithms, a set of common molecules are always detected as outliers: 22, 56, 58, 59, 60 and 67, and other molecules are detected as outliers only by one of both algorithms.

**Figure 2:**
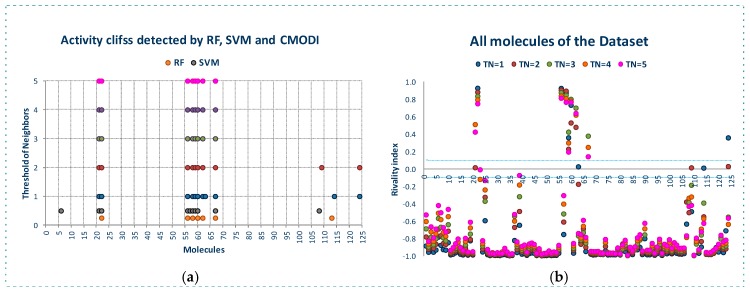
For YES1 dataset, outliers detected by the modelability index for different values of *TN* (**a**), values of the rivality index for different values of *TN* (**b**).

Figure 2 shows the behavior of the modelability and rivality indexes for YES1 dataset for values of threshold of neighbors (*TN*) from 1 to 5. As observed, *CMODI* index detects as outliers/activity cliffs practically the same molecules detected by RF and SVM algorithms (in Figure 2a are also represented the results for RF and SVM in the building of a model using CV).

We can appreciate in Figure 2a that the number of outliers detected by *CMODI* decreases as *TN* increases, although for any value of *TN* the molecules 22, 56, 58, 59 and 60 are detected as activity cliffs. Moreover, *CMODI* detects the molecules 62 and 67 as outliers, just as RF algorithm does. In addition *CMODI* also detects as an outlier the molecule 21, just as SVM does for some of the CV models built (see Figure 1).

Besides, we can observe in Figure 2a that the number of outliers detected by *CMODI* decreases as *TN* increases. Thus, molecules 114 and 124 only are detected as outliers for values of *TN* lower than 3. 

This behavior is clearly described in Figure 2b. In this figure we can observe the changes in the values of the rivality index for each molecule of the dataset with the changes in the *TN* values. For a high number of molecules, few changes in the *RI* values are observed. These molecules maintain values of *RI* very close to −1 and, therefore, these molecules are considered as correctly classifiable. As it can also be observed, these molecules are the same that RF and SVM algorithms classify correctly.

In addition, we can appreciate in Figure 2b that some molecules always have a positive value of the *RI* independently of the *TN* value. These molecules are the same molecules detected by RF and SVM algorithms as outliers for any CV and LOO model.

However, some molecules as 114 and 124 change from positive to negative their *RI* value with the increasing of *TN*. Thus, for values of *TN* greater than 2, *CMODI* considers these molecules as correctly classifiable, and we can check that RF and SVM classify these molecules correctly.

Moreover, we can interpret the deviation existing between the molecules detected as outliers by the algorithms and *CMODI*. For instance, the molecule 113 is detected as an outlier by RF algorithm (see also Figure 1). However, *CMODI* detects this molecule as correctly classifiable. Observing Figure 2b we see that this molecule is an activity border [26]. The molecule has a negative value very close to zero for low values of *TN*. When, *TN* increases the *RI* value for this molecule takes lower values and, therefore, is considered as correctly classifiable. Similar interpretation can be given for the molecules 108 and 124.

For the molecule 21, the opposite happens. This molecule is detected as an outlier by *CMODI* but the algorithms classify this molecule correctly (see also Figure 1). Observing the Figure 2b, this molecule is also an activity border, taking positive values very close to zero for low values of *TN*, and increasing the *RI* value when *TN* also increases.

**Figure 3:**
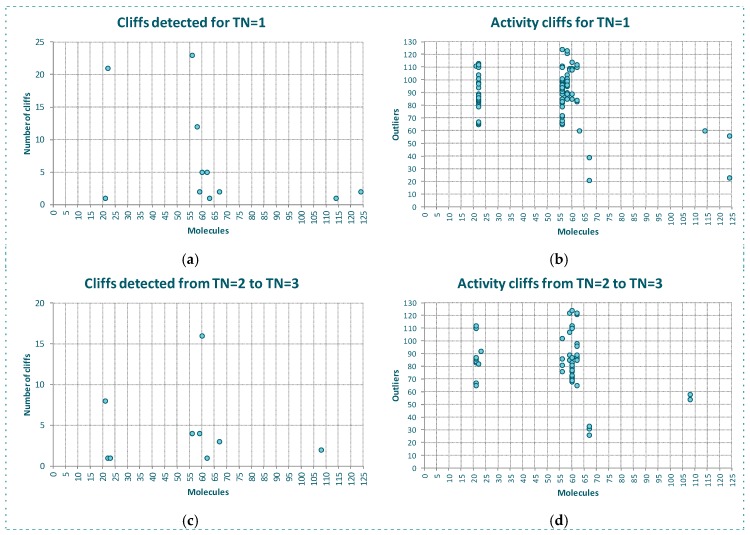
Study of the outlier behavior for YES1 dataset: (**a**,**c**) number nearest neighbors of a different class for the molecules of the dataset, (**b**,**d**) pairs of activity cliffs molecules.

The behavior of the outlier for YES1 dataset is even better shown in Figure 3. In Figure 3a we see, for each molecule of the dataset, the number of the firsts nearest neighbors belonging to a different class. As observed, the same molecules detected as outliers by the algorithms and *CMODI* have a high number of first nearest neighbors belonging to a different class closer than those belonging to the same class. 

For instance, there are 23 molecules of class 1 closer to the molecule 56 (class 0) than any other molecule of the dataset belonging to the class 0 (see Figure 3a). Thus, the molecule 56 is more similar to molecules belonging to class 1 than to any molecule belonging to class 0 and, therefore, this molecule is an activity cliff and it always will be detected as an outlier.

For other molecules, such as 21 and 114, there is only one closer molecule belonging to a different class. Consequently, if values of *TN* greater than 1 are considered these molecules could move from incorrectly to correctly classifiable.

Figure 3b shows this information in detail. Observing the X-axis, we see the molecules having their firsts nearest neighbor belonging to a different class. Thus, for instance, the first nearest neighbor of the molecule 21, above commented, is the molecule 110, and for the molecule 114 it is the molecule 60. We can also observe the 23 molecules belonging to the class 1 closer to the molecule 56 than any other molecule belonging to the class 0.

One of these molecules is the molecule 85. Now, observing the Y-axis, we can appreciate that this molecule 85 is also the first nearest neighbor of three molecules 22, 56, 58 and 60 and, therefore, this molecule 85 is the one responsible for these molecules being detected as activity cliffs.

In Figure 3c,d we have represented the same information but considering the behavior of the dataset for a neighborhood between *TN* = 2 and *TN* = 3. That is, only the presence of molecules between the second and the third neighbor of any class for each molecule of the dataset.

We observe that some molecules continue being affected by closer neighbors belonging to a different class. For instance, the molecule 60 will always be detected as outlier, because there are 16 molecules belonging to the class 1 closer than the third neighbor of the molecule 60 belonging to the class 0. However, this molecule 60 is not responsible for any other molecule to be detected as outlier, as happened for *TN* = 1.

Moreover, we see as the molecule 85 continues affecting other molecules of the dataset. For *TN* = 3, the molecule 85 affects to molecules 59 and 62 and, therefore, these molecules are detected as outliers by *CMODI* and the algorithms.

On the other hand, we can observe that the increasing of *TN* generates a diminishing of the number of molecules affecting the neighborhood of molecules of the same class to each molecule. Therefore, it produces a diminishing in the number of activity cliffs.

**Figure 4:**
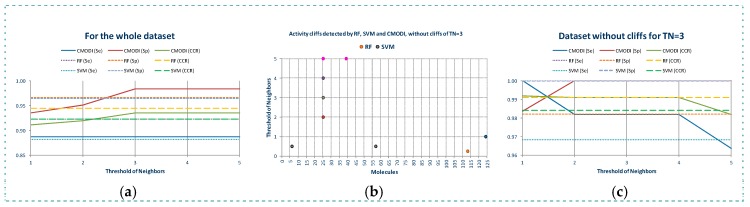
Modelability values and statistic parameter of the classification models built with RF and SVM algorithms for the whole dataset (**a**), outliers detected for the cleaned dataset without the activity cliffs detected for *TN* = 3 (**b**), and the resulting values of modelability and statistics parameters for the cleaned dataset without activity cliffs (**c**).

In Figure 4a are represented the results of the predictions generated using *CMODI* index and the statistics results obtained with RF and SVM algorithms, and in Table 1 are shown the values of the classification parameters.

As it can be observed, the values of the statistics increase as *TN* increases until a value of *TN* equal to 3. Thus, for YES1 dataset the consideration of the third nearest neighbors is enough to predict the behavior of the molecules of the dataset. The values obtained for the sensitivity, specificity and accuracy using the rivality index are very close to the values of these parameters obtained by the classification models generated with RF and SVM algorithms. We observe that the lower values are obtained for the sensitivity. As previously described, the higher number of activity cliffs are detected for the molecules belonging to class 0.

If we erase these activity cliffs detected for the dataset for a *TN* value equal to 3, we observe in Figure 4b that for *TN* = 1 only the molecule 124 is detected as an activity cliff by the rivality index, although for values of *TN* greater than 1, the molecule 26 is detected as an activity cliff. And for *TN* = 5 also the molecule 39. RF algorithm detects the molecule 113 and SVM algorithm the molecules 7 and 57 in the model built using CV.

Thus, as observed in Figure 4c and Table 1, by erasing the activity cliffs detected for the whole dataset and *TN* = 3, a clear improvement of the statistics parameters is obtained. Now, the prediction of *CMODI* and the *CCR* values for both algorithms are very close to 1, being the predictions obtained using the rivality index an excellent predictive parameter of the behavior of the classification algorithms of YES1 dataset. 

### 2.2. Analysis of the Applicability Domain of the Classification Models for the YES1 Dataset

With the aim of confirming the good behavior of the rivality index for the study of the applicability domain of the classification models, we have carried out the following experimental:

- YES1 dataset was randomly partitioned (80/20). This process was performed three times obtaining three sets for training (*TRS*) and three sets for external validations (*TES*). Thus, we can reproduce the behavior of the algorithms, the rivality and modelability indexes in the external validation process of the classification models, and to study the applicability domain of those models.
- Using the training sets (*TRS*), the classification models were built using RF and SVM algorithms and LOO technique. In this way, the results are reproducible and they are independent of the partitions generated when CV technique is used. For each of the models built, the values of sensitivity (*SE*), specificity (*SP*), accuracy (*ACC*) and *CCR* were stored.
- Values of the rivality index and *CMODI* were calculated for each one of the three *TRS*, and the activity cliffs detected were also stored.
- External validations were performed for each one of the three *TES* for each of the models built using RF and SVM.
- Finally, the analysis of the AD for the three *TES* was performed.

The analysis of the AD is carried out obtaining the values of the rivality index for each one of the molecules of the *TES* regarding the molecules of the corresponding *TRS*. This iterative procedure consists on adding one by one the molecules of the *TES* to the *TRS* and calculating the rivality index of the added molecule. Thus, the calculation of the AD is quite similar to the external validation process performed using the algorithms. 

**Figure 5:**
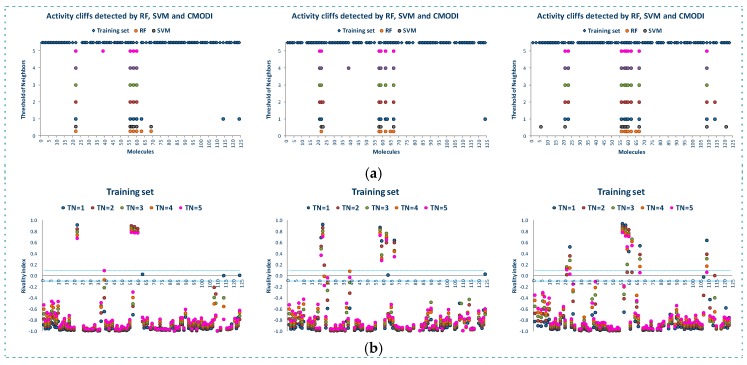
Outliers detected by the algorithms and activity cliffs detected by the rivality index for different values of *TN* for the three training sets (TRS) (**a**), values of the rivality index for the molecules included in the three training sets (**b**).

Figure 5 shows the results of the study of the AD for the three training sets. We observe that in spite of the different composition of the three training sets, the rivality index is capable of detecting the activity cliffs that the algorithms will detect as outliers. 

For a value of *TN* = 3, as described above, most of the outliers detected by RF and SVM in the building of the training models are detected by the *RI*, and any deviation is produced by those molecules described as activity borders, that is, those molecules with value of their rivality index close to zero (positive or negative).

This predictive capacity of the rivality index can be observed in Table 2. For the three training sets the values of *SE*, *SP*, *ACC* and *CMODI* obtained are very close to the corresponding values obtained in the building of the classification models using RF and SVM algorithms.

Therefore, the rivality index is capable of detecting those molecules of the datasets out of the AD. These molecules, those with high values of their rivality index, are detected as activity cliffs. These molecules introduce a non convenient noise in the training model, distorting the predictive robustness of that model to be used with an external (test) set.

**Figure 6:**
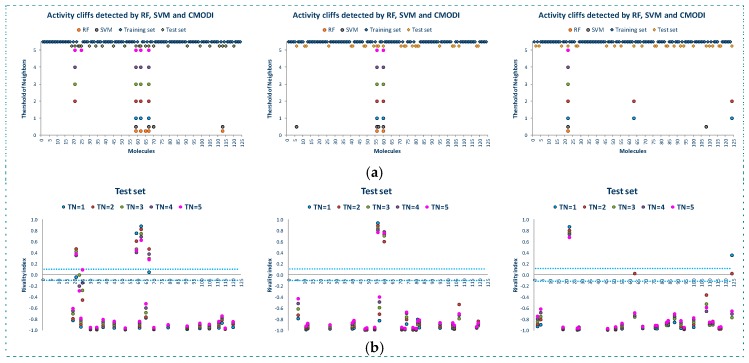
False positive/negative detected by RF and SVM algorithms and *CMODI* in the three external validations (**a**), rivality index values of the molecules of the external sets in the three external validations (**b**).

Figure 6 shows the results of the external validation carried out with the three test sets corresponding to the three training sets. As observed in this figure, the rivality index is an excellent predictive index of the behavior of the molecules of the test set. Independently of the composition of the training and test set, the rivality index detects those molecules that will be detected by the algorithms as false positive or false negative.

As observed in Figure 6b, these molecules have a high positive value of the rivality index and, therefore, the classification model built with the training set is out of the applicability domain to correctly predict the activity of these molecules included in the test set.

Observing the molecules incorrectly predicted by the algorithms and also detected as out of the AD by the rivality index (see Figure 6a, these molecules are the same to those detected as activity cliffs in the study of the whole dataset (see also Figure 2 and Figure 3). Therefore, these molecules are noisy points in any set, generating a distortion of any training model built including these molecules, and being always classified as false positive/negative in any external validation.

Once again, any deviation between the results obtained using the rivality index and the algorithms is caused by the activity borders. These type of molecules are in limit conditions to be correctly predicted and, therefore, the composition of the training set is determinant for their correct prediction. Thus, the simple and fast calculation of the rivality index for the molecules of the test set allows to the researcher to know the AD of the training model and the capacity of the building of a classification model to correctly predict the activity of each one of the molecules of the test set in the external validation process.

Table 3 shows the results of the three external validations carried out. As observed, in all cases the use of the rivality index and *CMODI* allows to obtain an excellent prediction of the results obtained using RF or SVM algorithms. All the statistics parameters obtained have very close or equal values to their corresponding parameter values obtained using RF or SVM.

### 2.3. Application to Datasets with Low Modelability

In the analysis and the results obtained, described above, we have applied our study to a dataset with high modelability. YES1 is a dataset containing a set of molecules perfectly distributed in two classes. Except for very few activity cliffs detected and described previously, the molecules of each class are very similar between each other, and the dissimilarity between molecules of different classes is high.

Regrettably, this characteristic is not common for most of the datasets. MRP4 is also a binary dataset with a number of molecules similar to YES1, but with a low modelability, having described a modelability of reference equal to 0.67 [27].

Thus, with this dataset we have carried out the same study to the one described for YES1 dataset, in order to demonstrate that the use of the rivality index for the analysis of the applicability domain of the classification models is extensible to any type of dataset.

**Figure 7:**
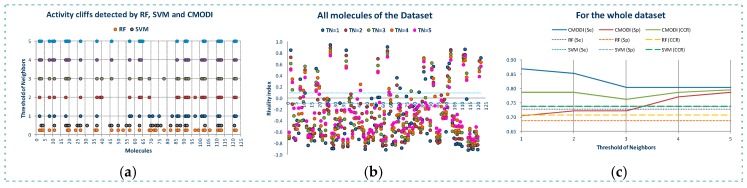
Activity cliffs detected by *CMODI* and outliers detected by RF and SVM algorithms (**a**), values of the rivality index for different values of *TN* (**b**), modelability values obtained by *CMODI* and the algorithms (**c**).

Figure 7a shows the reasons for the low modelability of MRP4 dataset. As observed, RF and SVM detect a lot of outliers belonging to both classes of molecules. Most of these outliers are also detected as activity cliffs by *CMODI* for different values of *TN*, having values of the rivality index greater than zero (see Figure 7b).

However, we can also observe in Figure 7b, the high number of molecules of this dataset having a value of the rivality index close to zero (positive or negative). Many of these activity borders are little affected by the value of the *TN*, and other activity borders change their rivality index value from negative to positive.

Thus, as it is represented in Figure 7c, higher values of *SE*, *SP* and *CCR* are obtained using *CMODI* than the ones obtained by the algorithms, because RF and SVM algorithms consider these activity borders as outliers (for instance, molecules 24 and 25). 

**Figure 8:**
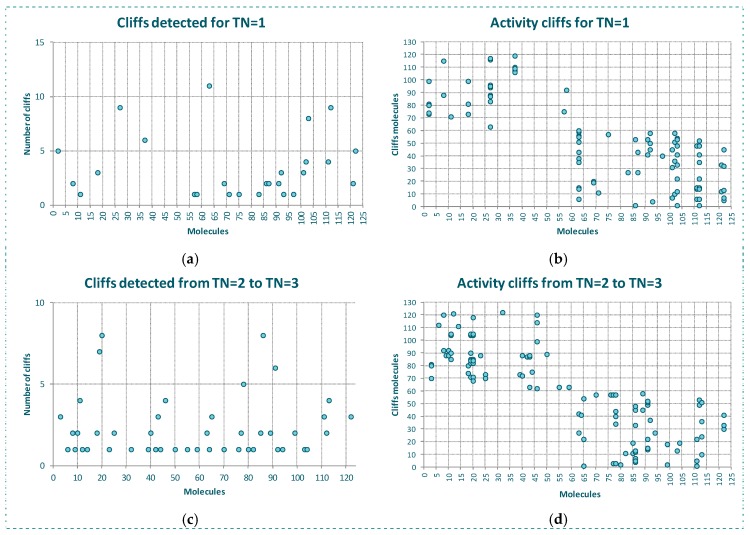
Study of the outlier behavior for MRP4 dataset: (**a**) number nearest neighbors of a different class for the molecules of the dataset for *TN* = 1, (**b**) pairs of activity cliffs for *TN* = 1, (**c**) number nearest neighbors of a different class for the molecules of the dataset for *TN* = 2 to *TN* = 3*,* (**d**) pairs of activity cliffs for *TN* = 2 to *TN* = 3.

These differences between YES1 and MRP4 datasets are clearly detected by the rivality index. Observing the Figure 8a,b, we can appreciate the higher number of molecules of MRP4 dataset having their firsts nearest neighbors belonging to a different class (see Figure 8a). For a lot of molecules their firsts nearest neighbor belongs to a different class and for other high number of molecules also the second and third nearest neighbors belong to a different class (see Figure 8c,d).

Thus, as we can observe in Figure 8b, in MRP4 dataset a lot of molecules are more similar to molecules of a different class than to any other molecule that belongs to its same class. For instance, the molecule 63 belongs to class 1, and it is more similar to 11 molecules (6, 14, 15, 35, 38, 43, 51, 55, 56, 59 and 60) belonging to class 0 than to any other molecule belonging to class 1. As a result, this molecule 63 is well detected by the rivality index as activity cliff, being considered as a molecule incorrectly classifiable by an algorithm. This prediction is confirmed by RF and SVM.

Due to the existence in MRP4 dataset of a lot of molecules having many nearest neighbors belonging to a different class, the consideration of values of *TN* greater than 1 will only affect slightly to the modelability of the dataset. Thus, as observed in Figure 8c,d for *TN* = 3, also a high number of molecules have a lot of nearest neighbors belonging to a different class closer than their third neighbor belonging to their same class (i.e., the molecule 20 has 8 molecules of the class 1 closer than its third first nearest neighbor belong to the class 0).

**Figure 9:**
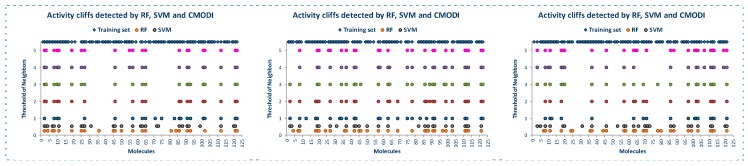
False positive/negative detected by RF and SVM algorithms and *CMODI* for the training sets in the three external validations carried out.

This problematic composition of molecules of MRP4 dataset is highlighted in the results of the three models built for the training sets in the three external validations performed with this dataset. As observed in Figure 9, independently of the training set composition, RF and SVM algorithms detect a lot of molecules as false positive/negative. Most of these molecules are also detected as activity cliffs by the rivality index, and the difference, as above explained, is due to the activity borders molecules (see Figure 9). Therefore, these results set in question the applicability domain of these models.

**Figure 10:**
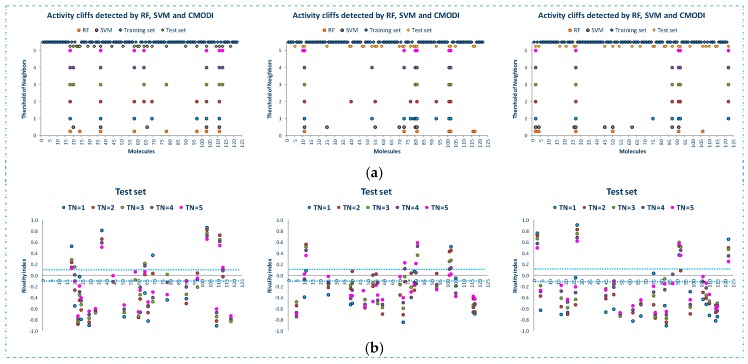
False positive/negative detected by RF and SVM algorithms and *CMODI* in the three external validations (**a**), rivality index values of the molecules of the external sets in the three external validations (**b**).

This suspect is confirmed by the results obtained in the three external validations. These results are shown in Figure 10 and we can observe a lot of molecules incorrectly predicted by the algorithms in the three internal models built.

These molecules belonging to the three test sets take values of the rivality index greater than zero for any value of *TN*. That is, in the training set there is always more than one nearest neighbor to any neighborhood value belonging to a different class. Or, in the case of the activity borders, in the training set there are molecules belonging to a different class very similar between each other and very similar to the molecules of the test set.

Thus, the modelability of the dataset is little affected by the value of the cardinality of the neighborhood (*TN*). That is due to the absence of a clear dissimilarity between molecules of the two classes, and as a result it is highly frequent that a molecule of a given class is more similar to its first, second, etc., neighbor of a different class than to another of the same class. The results of the internal model (for the training set) and external validation (for the test set) obtained for the three external validation carried out have shown these characteristics of the MRP4 dataset.

In Table 4 are shown the statistic values obtained for the internal models generated for the three training sets. We can observe that the values of *CMODI* are quite similar to the values of *CCR* corresponding with the models generated with RF and SVM algorithms for the whole training sets. The low modelability of this dataset is mainly caused by the molecules belonging to class 1 (molecules from 62 to 122), generating low values of the specificity. This fact was observed in the previously described analysis of the activity cliffs. For these molecules there is a high number of other more similar molecules belonging to class 0.

When, in the training sets, we erase the activity cliffs detected for a value of *TN* = 3, the new internal models built for the three training sets improve considerably. As shown in Table 4, the statistic parameters reach values greater than 0.9. Thus, as most of the activity cliffs for *TN* = 3 belong to the class 1, when erasing these molecules from the training sets, the specificity reaches values greater than 0.9. As observed, the values of the sensitivity also increase, but this increasing is very low due to the fact that few activity cliffs corresponding to class 0 have been erased.

However, although the training models improve when erasing the activity cliffs, the applicability domain of those training models does not improve. This behavior is observed analyzing the Table 5. In this Table 5 are shown the results of the three external validations carried out. We can observe how the values of the statistic parameters are highly affected by the composition of the training and test sets. 

Thus, for instance, using RF algorithm we have obtained values of the sensitivity of 0.69, 0.92 and 0.56 for each one of the three external validations using the whole training sets. If the activity cliffs for *TN* = 3 are erased from those training sets, values for the sensitivity of 0.39, 0.92 and 0.56, respectively, are obtained.

As a result, any model built with this dataset will have a low domain of applicability. As commented above, in this dataset there is no presence of a marked structural difference between molecules of both classes and, therefore, the prediction of the activity of an external molecule is clearly dependent of the molecules existing in the training for the building of the internal model.

We can also observe in Table 4 and Table 5 that, as above commented, the consideration of distinct values of *TN* has a very low effect in the results. Depending of the composition of the training and test sets, the best results are obtained for values of *TN* equal or close to 3. However, due to the fact that most molecules have nearest neighbors belonging to a different class at different values of *TN*, the changes in the neighborhood cardinality do not clearly improve the modelability of the dataset.

## 3. Discussion

The construction of robust and applicable classification models for datasets of molecules is a complex task. In this process, using techniques such as cross-validation, the modeler tests different algorithms by adjusting their parameters, in order to obtain a model with the higher reliability possible, so as to be used in the prediction of new molecules.

Independently of the mathematical basis of the algorithm used, all the algorithms build a classification model taking into account the similarity principle. Consequently, the presence in the dataset of activity cliffs, very similar molecules belonging to different classes, causes the detection of outliers by the classification model. As a result, classification models with low accuracy are obtained.

In the prediction of new molecules using the built classification model, these activity cliffs are considered and, moreover, new activity cliffs could be detected, generating incorrect predictions of the external molecules. 

In this paper we have described the capacity of the weighted rivality index to detect the activity cliffs present in the datasets. These molecules, detected as outliers by most of the algorithms, take positive values of the rivality index.

Consequently, the value of the rivality index corresponding to the molecules of the dataset allows the modeler to know the dataset characteristics in a previous stage to the construction of the classification model and the capacity of any algorithm to generate a robust classification model.

The values of the rivality index of the molecules of the dataset also allow to obtain a predictive value of the modelability of the dataset, being this value highly correlated with the correct classification rate value that will be obtained in the classification model generated by the algorithm.

Moreover, the study of the values of the rivality index considering different values of the neighborhood also allows the modeler to know the capacity of each molecule of the dataset to be correctly predicted by an algorithm.

Thus, by means of an easy and fast calculation, the rivality index allows to detect those molecules that will, always or depending on the consideration of a set of neighbors, be detected as outliers. 

As a result, this information will be used by the modeler for the depuration of the dataset in order to improve the robustness of the classification model.

In addition, in the prediction of new molecules, the rivality index offers an extra information to the modeler. Highly negative values of the rivality index will inform the modeler that a classification model built with the dataset is inside the applicability domain of prediction of those molecules.

On the contrary, if the external molecules take values of the rivality index close to one, the molecules of the dataset are outside of the applicability domain of prediction of those new molecules. Therefore, those molecules will, with a high probability, be predicted incorrectly.

In this paper, we have proved the capacity of the rivality index to inform the modeler about these characteristics of the dataset providing, in a stage previous to the building of the classification model by means of a fast calculation, information about the outliers and allowing the improvement of the robustness and applicability of the classification model.

The results obtained for a dataset of very high modelability and a dataset with very low modelability have shown that the proposal is applicable for any type of dataset, producing in any case an improvement of the accuracy of the classification models built and, moreover, a very clear improvement in the prediction of new molecules using those models.

## 4. Materials and Methods 

### 4.1. Datasets Description and Representation

From Chembench website [27], YES1 and 342_MRP4x (MRP4) datasets were gathered for the analysis and validation of the proposal presented in this paper. These datasets have been selected due to the fact that they contain a medium/low number of molecules, which will allow us to clearly show the graphics corresponding to the analysis carried out. 

YES1 and MRP4 are binary and perfectly balanced datasets. YES1 contains 124 molecules (1–62 belong to class 0 and 63–124 belong to class 1). MRP4 contains 122 molecules (1–61 belong to class 0, and 62–122 belong to class 1). However, the characteristics of these datasets are quite different. YES1 is a highly modelable dataset, being reported for this dataset a modelability index of reference equal to 0.85 [27]. On the contrary, MRP4 is a poorly modelable dataset, having a modelability index of reference equal to 0.67 [27].

The data representation of the datasets used in the calculations has been their descriptor matrices, also gathered from Chembench website [27]. The type of descriptor matrix selected has been CDK descriptors [28], containing 149 variables describing 1D and 2D molecular descriptors (see Supplementary Materials). 

### 4.2. Experimental Method

The descriptors matrix data representation of each dataset was analyzed, and columns (variables) having any value equal to NaN/Inf were erased. In addition, columns with value equal to zero for all rows were also deleted. The resulting matrices were normalized (range-scaled) by columns. Thus, maximum and minimum value of each column was calculated and Max/Min criterion was used to update the values of the matrix to their normalized values in the range [0, 1]. These normalized matrices were used in the calculations of the classification models and the indexes described in this paper.

For the calculation of the nearest neighbors, Euclidean distance was considered, and distance matrices were generated for each dataset. These matrices are symmetrical matrices of size *MxM*, being *M* the number of molecules of the dataset, where the elements (*i, j*) store the Euclidean distance between the molecules *i* and *j*. This Euclidean distance has been calculated using the range scaled descriptor matrix.

The diagonal of these matrices was set to *Inf*, and the distance matrices were sorted in ascending order by rows. Then, for each row (molecule) the neighbors to each molecule were ordered from the nearest (lower distance value) to the furthest (greater distance value).

For the calculations performed in this work, Matlab2017b [29] was used. In addition, using the Statistic and Machine Learning Toolbox [30], Support Vector Machine (SVM) with linear kernel, and ensemble bagged decision trees (Random Forest) algorithms were applied for the building of the classification models and calculation of the sensibility (*SE*), specificity (*SP*), accuracy (*ACC*) and correct classification rate (*CCR*). Ballabio et al. [31] have studied different metrics for the assessment of the classification performance, proving the excellent behavior of the sensitivity, specificity, accuracy and *CCR* in the analysis of classification results for binary and balanced datasets. In order to allow the experiments to be easily reproduced by other researchers, the algorithms were executed using the default values of the parameters established by Matlab2017b [29,30], so no changes were introduced for improving the results in the building of the classification models for the two datasets.

The building of the classification models was performed using Leave-One-Out (LOO) and 5-folds cross-validation (CV), with random partitioning of the dataset in the training set (80% of molecules) and test set (20% of molecules).

### 4.3. Rivality Index

The rivality index (*RI*) is a measurement of the capability to correctly predict the activity of a molecule by a statistic algorithm. For any molecule of a given dataset, the rivality index is defined as follows:

(3) $$\;RI_{i}=\frac{d_{i}^{x}-d_{i}^{y}}{d_{i}^{x}+d_{i}^{y}}\;$$

where: $d_{i}^{x}$ is the distance between the molecule *i* and its nearest neighbor molecule belonging to the same class of *i*, and $d_{i}^{y}$ is the distance between the molecule *i* and its nearest neighbor molecule that belongs to any class different to the class of molecule *i*.

The implementation of Equation (3) is quite simple. The Euclidean distances between all pairs of molecules of the dataset are obtained, and for each molecule the distance to the remaining molecules is sorted (this process is performed in the pre-processing stage as described above). When that is done it is only necessary to find, for each molecule, the first nearest neighbor of the same and of a different class.

*RI* is a normalized index which takes values between +1 and −1. Thus, values lesser than zero imply that the first nearest neighbor of molecule *i* is a molecule that belongs to the same class of *i,* and values of *RI* greater than zero mean that the first nearest neighbor of the molecule *i* is a molecule belonging to a different class of *i*.

*RI* values close to 1 mean that the first nearest neighbor belongs to the same class of a given molecule *i* is at a higher distance than any other molecule belonging to a different class. That is, there exists a molecule belonging to a different class much more similar to the given molecule *i* than any other molecule belonging to the same class of *i*.

Thus, the ratio between the summation of the values of *RI* for each class and the number of molecules of that class originates a global *RI*, informing of a global measurement of the modelability of the class, that is, the sensitivity and specificity values in the case of binary datasets. Besides, the summation of the global *RI* of all the classes in the dataset produces an absolute measurement of the dataset modelability. Global values of the *RI* for the dataset close to −1 imply that the data set is rather highly modelable. When values of *RI* for the dataset tend to zero, the modelability of the dataset decreases, and datasets with values of *RI* greater than zero are very little modelable.

### 4.4. Weighted Rivality Index

The rivality index is supported on the basis of QSAR approaches, considering that similar molecules should show similar properties. Therefore, if the first nearest neighbor to a given molecule belongs to the same class, that molecule should be correctly classified, and vice versa, if the first nearest neighbor of a given molecule belongs to a different class that molecule may be considered as an activity cliff. The molecule will have a positive value of its rivality index, being prognosticated as incorrectly classified by an algorithm.

However, there are molecules with values of *RI* equal to zero or very close to zero (positive or negative), being lower than the standard deviation of the distance between all pairs of molecules of the dataset. These molecules have been defined as activity borders [26].

Thus, in some cases, the numerator of Equation (3) takes the value $d_{i}^{x}$ = $d_{i}^{y}$ ≠ 0. These cases *RI* = 0, which informs about molecules being at the same distance of a molecule of its same class and another molecule of a different class. In other cases the numerator of Equation (3) takes the value $d_{i}^{x}$ = $d_{i}^{y}$ = 0. Then, in the dataset there exists at least three molecules (*i*, *x* and *y*) at the same distance and, therefore the value of $RI=\frac{d_{i}^{x}-d_{i}^{y}}{d_{i}^{x}+d_{i}^{y}}=\frac{0}{0}=NaN$ (indetermination). In this case, *RI* value is set to −10^−6^, in order to be differentiated of those cases in which $d_{i}^{x}$ = $d_{i}^{y}$ ≠ 0 and this term takes the value of 0.

We observe that this index takes into account only the first nearest neighbor of each molecule, not considering other nearest and similar molecules. In many cases, the first two neighbors of a molecule are molecules of either the same or different classes, but a considerable number of the following neighbors belong to a unique class (equal or different to the one of the considered molecule). Thus, when some of the *N* firsts neighbors are selected in the training set, a different result is obtained than when some of these molecules are not in the training set.

Thus, we defined a weighted rivality index as follows:

(4) $$\;RI=\frac{\left(d_{i}^{x}*w_{i}^{x})-(d_{i}^{y}*w_{i}^{y}\right)}{\left(d_{i}^{x}*w_{i}^{x})+(d_{i}^{y}*w_{i}^{y}\right)}\;$$

where: $d_{i}^{x}$ is the Euclidean distance from the molecule *i* to its first nearest neighbor *x* belonging to the same class of *i*, $d_{i}^{y}$ is the Euclidean distance from the molecule *i* to its first nearest neighbor *y* that belongs to a different class than the one of *i*, $w_{i}^{x}$ is the weight assigned to the neighbor that belongs to the same class of *i* and $w_{i}^{y}$ is the weight assigned to the neighbor belonging to a class different to the class of *i*.

These weights are calculated through the following expression:

(5) $$\;w_{i}^{x}=\frac{CN_{i}-CN_{i}^{x}}{CN_{i}},\;w_{i}^{y}=\frac{CN_{i}-CN_{i}^{y}}{CN_{i}}\;$$

where: $w_{i}^{x}$ is the weight assigned to the distance between the molecule *i* and its first nearest neighbor *x* of the same class of *i*, $w_{i}^{y}$ is the weight assigned to the distance between the molecule *i* and its first nearest neighbor *y* of a different class than the one of *i*, *CN_i_* is the cardinality of the neighborhood assigned to the molecule *i*, $CN_{i}^{x}$ and $CN_{i}^{y}$ are the cardinalities in the neighborhood of the molecules belonging to the same and different classes of molecule *i*, respectively. 

The value of the cardinality of the neighborhood is calculated using a threshold of neighbors (*TN*) describing the minimum number of neighbors of each class that must exist in the neighborhood. Thus, selecting a *TN* for the dataset, the cardinality of the neighborhood could be different for each molecule due to the fact that, for each molecule *i*, its *TN* first nearest neighbors of each class can be in a different order depending on their distance to that molecule *i*.

As the value of *TN* changes, the cardinality of the neighborhood also changes, and this value of the cardinality of the neighborhood could be different for each molecule of the dataset. Thus, considering this threshold (*TN*) for the calculation of the *CN_i_* value, we can partially estimate that the status of the neighborhood of each molecule of the dataset is like the one existing in for any partition of the dataset in the cross-validation stage.

### 4.5. Modelability Index

The rivality index is a DM measurement of the predictability of a molecule combining the CLASS-LAG [23] and population analysis methods [20] in a simpler definition of AD and in a fastest calculation. Thus, the calculation of *RI* does not require the building of a model, the values of AD contribute with an absolute measurement of the capability to a molecule to be correctly predicted by a model and, in addition, these *RI* values inform of the molecules inside, outside and borders of the AD.

High positive values of *RI* describe molecules outside of the AD, low negative values of *RI* describe molecules inside of the AD having a high predictability accuracy, and molecules with values (positive/negative) of *RI* close to zero describe molecules with high uncertainty to be predicted. However, the weighted of the *RI* considering the neighborhood of each molecule allows to refine this uncertainty, informing of those molecules existing in the training set responsible of the low accuracy of the predictability and allowing to improve the AD by means of the curation of the training set.

Therefore, values lower than zero of the weighted rivality index describe molecules capable of being correctly classified by an algorithm. That way, we can obtain a measurement of the dataset modelability. The MODelability Index (*CMODI*) [26] is a quantitative measurement to quickly assess whether a predictive QSAR model can be obtained for a chemical dataset and can be calculated as follows:

(6) $$\;CMODI=\frac{1}{k}\overset{k}{\underset{j=1}{\sum }}\left[\frac{1}{M_{k}}\overset{M_{k}}{\underset{i=1}{\sum }}1,\;\forall RI_{i}\leq 0\right]\;$$

where: *k* is the number of exiting classes in the dataset, *RI_i_* is the value of the rivality index of the molecule *i* and *M_k_* is the number of molecules of the class *k* in the dataset.

*CMODI* takes values between 0 and 1 and is a measure of the number of molecules of a dataset that will be correctly classified by an algorithm. Therefore, it is a predictive measurement of the value of the correct classification rate (*CCR*) obtained by an algorithm. In addition, analyzing the Equation (6), it can be observed that the expression between brackets calculates the sensibility/specificity, that is, the modelability of each class for binary datasets.

## Figures and Tables

**Table 1:** Results of the predictions using *CMODI* for different values of TN and the algorithms for the whole datasets and the dataset erasing the activity cliffs detected for *TN* = 3.

		All Molecules of the Dataset					Erasing Cliffs for *TN* = 3				
Algorithm	*TN*	*SE*	*SP*	*ACC*	*CMODI*	*CCR*	*SE*	*SP*	*ACC*	*CMODI*	*CCR*
	1	0.887	0.935	0.911	0.911		1.000	0.984	0.991	0.992	
	2	0.887	0.952	0.919	0.919		0.982	1.000	0.991	0.991	
	3	0.887	0.984	0.935	0.935		0.982	1.000	0.991	0.991	
	4	0.887	0.984	0.935	0.935		0.982	1.000	0.991	0.991	
	5	0.887	0.984	0.935	0.935		0.964	1.000	0.983	0.982	
RF		0.923	0.966	0.944		0.945	1.000	0.982	0.991		0.991
SVM		0.882	0.964	0.919		0.923	0.968	1.000	0.983		0.984

**Table 2:** Results of the predictions using *CMODI* for different values of *TN* and the algorithms for the three whole training sets and the training sets erasing the activity cliffs detected for *TN* = 3.

		All Molecules of the Dataset						Erasing Cliffs for *TN* = 3				
Algorithm	*TN*	*SE*	*SP*	*ACC*	*CMODI*	*CCR*		*SE*	*SP*	*ACC*	*CMODI*	*CCR*
		Training set TRS-01										
	1	0.918	0.941	0.930	0.930			1.000	1.000	1.000	1.000	
	2	0.918	1.000	0.960	0.959			1.000	1.000	1.000	1.000	
	3	0.918	1.000	0.960	0.959			1.000	1.000	1.000	1.000	
	4	0.918	1.000	0.960	0.959			1.000	1.000	1.000	1.000	
	5	0.898	1.000	0.950	0.949			0.978	1.000	0.990	0.989	
RF		0.925	0.957	0.940		0.941		1.000	1.000	1.000		1.000
SVM		0.909	0.978	0.940		0.943		0.980	0.978	0.979		0.979
		Training set TRS-02										
	1	0.900	0.939	0.919	0.919			1.000	0.979	0.989	0.990	
	2	0.880	0.980	0.929	0.930			0.978	1.000	0.989	0.989	
	3	0.900	0.980	0.939	0.940			0.978	1.000	0.989	0.989	
	4	0.880	0.980	0.929	0.930			0.978	1.000	0.989	0.989	
	5	0.900	0.980	0.939	0.940			0.978	1.000	0.989	0.989	
RF		0.922	0.958	0.939		0.940		1.000	0.978	0.989		0.989
SVM		0.923	0.979	0.949		0.951		1.000	1.000	1.000		1.000
		Training set TRS-03										
	1	0.868	0.935	0.899	0.901			1.000	1.000	1.000	1.000	
	2	0.868	0.935	0.899	0.901			1.000	1.000	1.000	1.000	
	3	0.868	0.957	0.909	0.912			0.978	1.000	0.989	0.989	
	4	0.868	0.957	0.909	0.912			0.978	1.000	0.989	0.989	
	5	0.868	0.957	0.909	0.912			0.957	1.000	0.978	0.978	
RF		0.898	0.960	0.929		0.929		1.000	0.979	0.989		0.989
SVM		0.860	0.939	0.899		0.899		0.957	1.000	0.978		0.978

**Table 3:** Results of the predictions using *CMODI* for different values of *TN* and the algorithms for the three whole training sets and the training sets erasing the activity cliffs detected for *TN* = 3.

		All Molecules of the Training Sets						Erasing Cliffs for *TN* = 3				
Algorithm	*TN*	*SE*	*SP*	*ACC*	*CMODI*	*CCR*		*SE*	*SP*	*ACC*	*CMODI*	*CCR*
		Test set TES-01										
	1	0.846	0.909	0.875	0.878			0.769	0.909	0.833	0.838	
	2	0.769	0.909	0.833	0.839			0.692	0.909	0.792	0.801	
	3	0.769	0.909	0.833	0.839			0.692	0.909	0.792	0.801	
	4	0.769	0.909	0.833	0.839			0.692	0.909	0.792	0.801	
	5	0.692	0.909	0.792	0.801			0.692	0.909	0.792	0.801	
RF		0.846	0.727	0.792		0.787		0.846	0.818	0.833		0.832
SVM		0.923	0.727	0.833		0.825		0.923	0.909	0.917		0.916
		Test set TES-02										
	1	0.833	1.000	0.920	0.917			0.833	1.000	0.917	0.920	
	2	0.833	1.000	0.920	0.917			0.833	1.000	0.917	0.920	
	3	0.833	1.000	0.920	0.917			0.833	1.000	0.917	0.920	
	4	0.833	1.000	0.920	0.917			0.833	1.000	0.917	0.920	
	5	0.833	1.000	0.920	0.917			0.833	1.000	0.917	0.920	
RF		0.833	1.000	0.920		0.917		0.833	1.000	0.920		0.917
SVM		0.667	1.000	0.840		0.833		0.667	1.000	0.840		0.833
		Test set TES-03										
	1	0.889	0.875	0.880	0.882			0.889	0.938	0.920	0.913	
	2	0.889	0.875	0.880	0.882			0.889	0.938	0.920	0.913	
	3	0.889	1.000	0.960	0.944			0.889	1.000	0.960	0.944	
	4	0.889	1.000	0.960	0.944			0.889	1.000	0.960	0.944	
	5	0.889	1.000	0.960	0.944			0.889	1.000	0.960	0.944	
RF		0.889	1.000	0.960		0.944		0.889	1.000	0.960		0.944
SVM		0.889	0.938	0.920		0.913		0.889	1.000	0.960		0.944

**Table 4:** Results of the MRP4 predictions using *CMODI* for different values of *TN* and the algorithms for the three whole training sets and the training sets erasing the activity cliffs detected for *TN* = 3.

		All Molecules of the Dataset						Erasing Cliffs for *TN* = 3				
Algorithm	*TN*	*SE*	*SP*	*ACC*	*CMODI*	*CCR*		*SE*	*SP*	*ACC*	*CMODI*	*CCR*
		Training set TRS-01										
	1	0.904	0.733	0.825	0.819			0.977	0.970	0.974	0.973	
	2	0.885	0.733	0.814	0.809			0.953	0.909	0.934	0.931	
	3	0.827	0.733	0.784	0.780			0.977	0.909	0.947	0.943	
	4	0.808	0.756	0.784	0.782			0.977	0.818	0.908	0.897	
	5	0.827	0.644	0.742	0.736			0.977	0.758	0.882	0.867	
RF		0.707	0.714	0.711		0.711		0.882	0.929	0.908		0.905
SVM		0.714	0.727	0.722		0.721		0.848	0.884	0.868		0.866
		Training set TRS-02										
	1	0.735	0.708	0.722	0.722			0.947	0.969	0.957	0.958	
	2	0.776	0.667	0.722	0.721			0.947	0.906	0.929	0.927	
	3	0.776	0.667	0.722	0.721			0.947	0.969	0.957	0.958	
	4	0.796	0.688	0.742	0.742			0.868	0.969	0.914	0.919	
	5	0.776	0.729	0.753	0.752			0.868	0.969	0.914	0.919	
RF		0.868	0.694	0.691		0.691		0.964	0.881	0.914		0.923
SVM		0.735	0.750	0.742		0.742		0.903	0.897	0.900		0.900
		Training set TRS-03										
	1	0.869	0.705	0.787	0.787			0.959	0.977	0.968	0.968	
	2	0.852	0.721	0.787	0.787			0.939	0.977	0.957	0.958	
	3	0.803	0.721	0.762	0.762			0.959	0.977	0.968	0.968	
	4	0.803	0.770	0.787	0.787			0.939	0.977	0.957	0.958	
	5	0.803	0.787	0.795	0.795			0.939	0.955	0.946	0.947	
RF		0.712	0.698	0.705		0.705		0.860	0.977	0.914		0.918
SVM		0.746	0.763	0.754		0.754		0.840	0.953	0.892		0.897

**Table 5:** Results of the MRP4 predictions using *CMODI* for different values of *TN* and the algorithms for the three whole training sets and the training sets erasing the activity cliffs detected for *TN* = 3.

		All Molecules of the Training Sets						Erasing Cliffs for *TN* = 3				
Algorithm	*TN*	*SE*	*SP*	*ACC*	*CMODI*	*CCR*		*SE*	*SP*	*ACC*	*CMODI*	*CCR*
		Test set TES-01										
	1	0.769	0.636	0.708	0.703			0.692	0.545	0.625	0.619	
	2	0.769	0.545	0.667	0.657			0.692	0.545	0.625	0.619	
	3	0.769	0.545	0.667	0.657			0.692	0.636	0.667	0.664	
	4	0.692	0.636	0.667	0.664			0.692	0.636	0.667	0.664	
	5	0.769	0.636	0.708	0.703			0.615	0.545	0.583	0.580	
RF		0.692	0.636	0.667		0.664		0.385	0.455	0.417		0.420
SVM		0.846	0.727	0.792		0.787		0.692	0.636	0.667		0.664
		Test set TES-02										
	1	0.833	0.385	0.600	0.609			0.667	0.692	0.680	0.679	
	2	0.750	0.615	0.680	0.683			0.667	0.769	0.720	0.718	
	3	0.917	0.682	0.800	0.804			0.583	0.615	0.600	0.599	
	4	0.833	0.615	0.720	0.724			0.583	0.538	0.560	0.561	
	5	0.833	0.615	0.720	0.724			0.583	0.538	0.560	0.561	
RF		0.917	0.538	0.720		0.728		0.917	0.615	0.760		0.766
SVM		0.750	0.692	0.720		0.721		0.750	0.692	0.720		0.721
		Test set TES-03										
	1	0.778	0.688	0.720	0.723			0.778	0.750	0.760	0.764	
	2	0.778	0.750	0.760	0.764			0.778	0.750	0.760	0.764	
	3	0.778	0.750	0.760	0.764			0.778	0.750	0.760	0.764	
	4	0.778	0.750	0.760	0.764			0.778	0.750	0.760	0.764	
	5	0.778	0.813	0.800	0.795			0.778	0.750	0.760	0.764	
RF		0.556	0.875	0.760		0.715		0.556	0.875	0.760		0.715
SVM		0.333	0.813	0.640		0.573		0.333	0.688	0.560		0.510

## Supplementary Materials

YES1.xlsx and 342_MRP4x.xlsx files containing the information of the datasets used in this manuscript are available online.
